# Facial mask personalization encourages facial mask wearing in times of COVID-19

**DOI:** 10.1038/s41598-021-04681-y

**Published:** 2022-01-18

**Authors:** Johanna Palcu, Martin Schreier, Chris Janiszewski

**Affiliations:** 1 Department of Marketing, Vienna University of Economics and Business (WU), Vienna, Austria; 2 Warrington College of Business, University of Florida, Gainesville, FL USA

**Keywords:** Human behaviour, Psychology, Diseases, Health policy, Public health

## Abstract

One of the most cost-effective strategies for fighting the spread of COVID-19 is the use of facial masks. Despite health officials’ strong efforts to communicate the importance or wearing a mask, compliance has been low in many countries. In the present paper we propose a novel behavior-intervention strategy to encourage people to wear facial masks. Three studies show that the personalization of a mask, as a form of identity expression, increases mask wearing intentions and, by extension, the percentage of individuals who wear facial masks. Given that mask wearing remains a necessity after deployment of the first vaccines, novel approaches to encouraging mask wearing are essential. Linking facial mask wearing to an individual’s identity is a promising strategy.

## Introduction

“The purpose of life is self-expression. Expressing your essence entirely is what we live for”. (Oscar Wilde).

COVID-19 is one of the biggest health-related challenges the planet has faced since the Black Plague (1347–1351). Governments and public health officials have taken several actions to fight the pandemic, including ordering lockdowns, mandating physical distancing, and requesting that citizens wear face masks and frequently disinfect their hands. Yet, these actions have been insufficient. The third, and largest, wave of the pandemic occurred in fall, 2020. Indeed, as of October 1, 2021, Johns Hopkins estimates COVID-19 had infected more than 230 million people and resulted in more than 4.8 million deaths^1^.

One of the most cost-effective strategies for fighting the spread of COVID-19 is the use of a facial mask in enclosed environments or when in close contact with others^2–6^. Facial masks reduce the amount of disease carrying particulates that infected people exhale into the air and uninfected people inhale into their lungs. In fact, US experts claim “… universal mask use (95% mask use in public) could be sufficient to ameliorate the worst effects of epidemic resurgences in many states”^7^, a claim that has been supported empirically in the general population^8^.

Governments and public health officials have framed the request to wear a mask in the classic textbook fashion: that is, they have stressed that compliance is a socially responsible behavior and that a lack of compliance will lead to negative consequences for the general public. Unfortunately, recent evidence suggests that emphasizing social responsibility in health messaging results in a small influence (i.e., less than 1% variance explained) on pandemic-fighting behaviors (e.g., washing hands, avoiding large gatherings, sharing public health messages with others)^9^. This conclusion is consistent with low mask wearing compliance in many European countries (Fig. S1, “Supplementary materials”)^10^, most of which have relied on public health campaigns that stress social responsibility.

Indeed, of all the preventative public health behaviors designed to control COVID-19, few have been as controversial and wildly debated as facial mask wearing. In June 2020, for example, the New York Times wrote that masks have become a “flash point in the virus culture wars”^11^. In France, anti-mask groups (“les anti-masques”) have used social media to call for an “end to the masquerade” (“Stop à la masque-arade”) and talked about the tyranny of a “health dictatorship”^12^. Spanish citizens have protested against wearing a mask and asked for “help in saving their freedom” (Ayudadnos a salvar la liberdad)^13^. In Germany, pictures of the anti-pandemic movement “Queerdenker” (English: “lateral thinker”) feature people without face masks.

A central reason for the variable, and sometimes low, compliance with face mask requests may lie in the very nature of the behavior. Wearing a face mask is a restriction and, as humans, we do not like being restricted. Moreover, the restrictions or “personal costs” that come with wearing a face mask are not only physical (i.e., not being able to breathe properly, facial discomfort) but also psychological (i.e., feeling disguised, de-individualized, or stigmatized)^14^. To address this issue, we propose a fundamentally different behavior-intervention strategy which is based on the insight that people comply with requests that are in line with their individual needs. In doing so, we respond to the recent call to focus on the psychological drivers of pandemic-fighting behaviors^15–17^.

This research demonstrates that mask wearing increases when the behavior is aligned with a person’s need for self-expression. Self-expression involves “expressing one's thoughts and feelings, and these expressions can be accomplished through words, choices, or actions”^18^. Reinforcing what Oscar Wilde argued more than 100 years ago (our opening quote), self-expression norms are currently “at an all-time high”^19^, p. 60. Notably, self-expression is often realized via consumption choices^20–22^, as such choices help people “make their preferences and values overt and observable”^22^, p. 1. Self-expression is also realized through product customization, an act that allows a product to reflect the owner’s identity and personality^23^. Indeed, prior research indicates that participating in the design or construction of a product increases the perceived value of the product^24–26^, enhances beliefs about the efficacy of the product^27^, and increases performance that relies on the use of the product^28^.

Against this background, we argue that if face masks help people express who they are, then individuals might gain more personal benefit from wearing masks. Derived from the literature linking personalization and behavior, we propose that imbuing individuality into masks will increase the percentage of people who wear a mask as well as the frequency of their use. We report three studies that test our prediction. Study 1 is a correlational study confirming a positive relationship between mask personalization and the frequency of mask usage in the general population. Study 2 uses an online experiment to provide causal evidence for the proposed effect. Finally, Study 3 reports the results of a field experiment conducted on a European university campus in between two lockdowns.

## Study 1

### Method

#### Ethics statement

According to the Austrian Universities Act 2002 (UG2002), which was in place during the execution of the present studies, only medical universities are required to appoint an ethics committee for clinical trials and medical research. Consequently, the conducting institution did not carry an institutional review board at the time of study and no specific ethical approval for the present studies was required. The present research was conducted in accordance with the Declaration of Helsinki (revised 1983) and the local code of conduct for scientific research of the Austrian University in charge of the research. Data was collected anonymously and in accordance with the most recent version of the European General Data Protection Regulation (2016/679). All participants gave informed consent prior to participation and could withdraw at any time during the experiment without further consequences.

#### Participants and design

To establish the connection between the individuality expressed by the mask and mask wearing, we surveyed a nationally representative sample of 1224 Austrian citizens (51.1% female, 48.9% male; *M*_age_ = 47.63, *SD*_age_ = 15.87) in May, 2020. The large-scale survey was conducted by a professional research agency and assessed consumer behaviors during the COVID-19 pandemic, including tracking app acceptance, changes in purchasing behavior, and (most important for this research) the self-reported frequency of mask usage. The questionnaire was administered in German.

#### Procedure and measures

The ad hoc questionnaire was administered online in two waves, with a 10-day time period in-between the waves (for an overview of the questions, see Tables S2–S4 in the “Supplementary materials”). Austria was in lockdown during this period (i.e., protective masks were mandatory in all stores, public buildings, and on public transportation).

The questionnaire began by reminding participants of the government’s current mask wearing policy: “Since the beginning of April wearing a protective mask is mandatory in all stores, in public buildings, and on public transportation. In the following study, we would like to know more about how you are personally affected by this regulation”. The critical outcome variable was then assessed by asking participants to indicate mask wearing frequency: “Please think about how often you have worn your mask over the past few days. Please indicate how frequently you have worn the mask”; 1 = only when I really had to (e.g., in the store or public transportation), 7 = as frequently as possible (e.g., when leaving the house). The critical predictor variable was the individuality expressed by the respondent’s mask. Individuality was assessed with one item: “Think about the mask that you wear most often. Where would you place that mask on a scale from a typical mask to a very individual mask?”; 1 = typical, 6 = individual)”. Two additional control measures, known to influence mask wearing, were collected. COVID-19 risk perception was assessed using three items: “I feel that the COVID-19 crisis is a severe threat”, “The current COVID-19 situation makes me feel worried”, “I feel that the crisis around COVID-19 is overblown” (r); 1 = totally disagree, 5 = totally agree; α = 0.82). The degree of restriction created by wearing the mask was assessed using three items: “I feel physically restricted when wearing the mask”, “With my mask, I feel that I am not ‘functioning’ properly (i.e., my senses are restricted)”, “When wearing the mask, I feel that I have everything under control (r)”; 1 = totally disagree, 7 = totally agree; α = 0.70). Other measures not relevant for the present research are reported in the “Supplementary materials” (Tables S2–S4).

### Results

Regression was used to assess the influence of the independent variables (mask individuality, COVID-19 risk perception, restrictiveness of the mask) on the dependent measure (recent frequency of mask usage). A partial r-square was calculated to assess the influence of each independent variable while controlling for the other two independent variables. People wore a mask more frequently when they perceived a mask to express their individuality (*B* = 0.107, *SE* = 0.029, *t* = 3.61, *p* < 0.001, *r*^2^_partial_ = 0.011) and when their risk perception was higher (*B* = 0.412, *SE* = 0.059, *t* = 7.002, *p* < 0.001, r^2^_partial_ = 0.039), but less frequently when they perceived a mask to be restrictive (*B* = -0.359, *SE* = 0.038, *t* = -3.61, *p* < 0.001, *r*^2^_partial_ = 0.060). Thus, the results of our first study indicate that the individuality expressed by a mask remained a significant predictor of mask wearing frequency even after controlling for COVID-19 risk perception and the perceived degree of physical restriction when wearing a mask.

## Study 2

### Method

#### Participants and design

The second study was designed to assess the direct influence of personalizing a mask on the intention to wear a mask. A convenience sample of 895 paid UK citizens, recruited through the online research platform Prolific (59.3% female, *M*_age_ = 38.71, *SD*_age_ = 13.59), responded to two surveys administered 3 days apart.

#### Procedure and measures

Data were collected in December 2020. The UK was in lockdown during that period (i.e., protective masks were mandatory in all stores, public buildings, and on public transportation). The survey was distributed online in two waves with a 3-day delay in between waves. During the first wave participants were asked to imagine purchasing a mask. All participants were then shown a stack of white masks and told to pick one of them. We assessed intended frequency of wearing the mask with one item: “How frequently do you think you will wear the mask that you just picked?”; 1 = only when really necessary (e.g., in public transportation or indoors), 7 = whenever I can (e.g., as soon as I leave the store).

The second wave consisted of a two-cell design: half of the participants repeated the first wave scenario. The other participants were instructed to imagine that they had the opportunity to personalize the mask according to their own taste. Participants indicated how their personalized mask would look (free response). For both groups, we then measured participants intended frequency of wearing the mask with the same item that was used during the first wave.

In addition, as a manipulation check, we assessed the extent to which the mask expressed individuality using two items: “Concerning the mask that you just picked: Please indicate how much you agree with the following statements: This mask allows me to express my identity”; “This mask helps me signal who I am”; 1 = totally disagree, 7 = totally agree; r = 0.92. Finally, participants indicated the perceived price of the mask (i.e., “This mask is…”; 1 = very cheap, 7 = very expensive) and expected physical restrictiveness of the mask (i.e., “I would feel physically restricted when wearing the mask”; 1 = totally disagree, 7 = totally agree). Other measures are reported in the “Supplementary materials” (Table S6).

### Results

The manipulation check indicated higher self-expressiveness in the personalized mask condition than in the control condition (*M*_control_ = 2.09, *SD* = 1.27; *M*_personalized_ = 4.72, *SD* = 1.84; *F*(1, 893) = 620.53, *p* < 0.001, ω2 = 0.41). The difference score in participants’ intended frequency of wearing the mask (wave 2 − wave 1) served as dependent variable. Results show that the opportunity to personalize the mask increased the intended frequency of wearing the mask (wave two − wave one) relative to the control group (Δ*M*_control_ = 0.08, *SD* = 1.43; Δ*M*_personalized_ = 0.47, *SD* = 1.82; *F*(1, 893) = 12.93, *p* < 0.001, ω^2^ = 0.01). Thus, the second study offers support for the direct link between the opportunity to self-express through a face mask (via the personalization of the mask) and the intended frequency of mask usage.

## Study 3

### Method

#### Participants and design

A field study was designed to test the relationship between mask personalization and mask wearing. Ninety-nine university students (73.3% female, *M*_age_ = 23.13, *SD*_age_ = 2.30) were recruited using campus flyers and were paid 10 Euro to complete a laboratory experiment. Participants were randomly assigned to one of two between-subjects conditions that differed in whether participants wore a plain mask or a personalized mask during an on-campus activity.

#### Procedure and measures

The data were collected in October, 2020 in Austria just before the second nationwide lockdown. Participants were invited into a university lab and, upon arrival, were told that they would participate in two separate studies. They were informed that the first study was about how people personalize masks and the second study involved a short mystery shopping task on campus. In the first study, participants were given a plain white mask, a variety of patches, colored markers, and stamps, and were asked to personalize it. Participants were given a short instruction on how to personalize their mask (e.g., “Try not to go crazy and “overdesign”. You should create something that you like and that reflects your personal style”; see Fig. S6 in the “Supplementary materials” for instructions) and some samples to look at (see Fig. S9 in the “Supplementary materials”). After personalizing the mask, they started the second study. In this study, they were told they would be a mystery shopper at the local bakery (a two-block walk from the lab). As a cover story participants were given two euros and asked to buy at least one product. They were also instructed to note the ambience of the bakery, look at all the available products, and pay attention to the service encounter. Participants in both conditions were then told they would wear a mask so that the conditions of the shopping trip were similar for everyone. In the control condition participants were then handed a clean, white mask before leaving the lab. Participants in the personalized mask condition were given the mask they had personalized. Before leaving the lab, all participants received information that mask wearing was mandatory indoors but voluntary when outside (see Fig. S7 in the ‘Supplementary materials” for instructions). Participants were unaware of the fact that the first and second study were related.

Research assistants, who were blind to the focal research hypothesis, were located between the lab and the bakery. They observed whether participants wore the mask on five different occasions: when they left the lab, when they arrived at the bakery, when they left the bakery, when they talked to a research assistant located outside the bakery, and when they arrived back at the lab. The dependent measure was whether the mask was worn for the entire trip (dummy coded).

Upon returning to the lab, participants were asked to fill out a short questionnaire. Participants indicated the extent to which wearing the mask was fun with one item: “I enjoyed wearing the mask”; 1 = totally disagree, 7 = totally agree. Moreover, we assessed the expressed individuality of the mask (2 items, *r* = 0.89), COVID risk perception (3 items, α = 0.60), and physical restrictiveness (1 item) with the same items as in our previous studies (see Table S8 in the “Supplementary materials”).

### Results

The manipulation check indicated higher levels of self-expressiveness when wearing the personalized mask rather than the plain white mask (*M*_control_ = 2.53, *SE* = 1.63; *M*_personalized_ = 3.73, *SE* = 1.61; *F*(1, 98) = 13.50, *p* < 0.001, ω^2^ = 0.11). The results show that personalizing the mask increased the likelihood of wearing the mask (p_control_ = 14.6%, *p*_individualized_ = 36%; Χ^2^ = 5.91, *p* = 0.015, φ = 0.24). We also found that the personalized mask was more fun to wear (*M*_control_ = 3.67, *SE* = 1.79; *M*_personalized_ = 4.75, *SE* = 1.92; *F*(1, 98) = 8.34, *p* < 0.001, ω^2^ = 0.07).

## Discussion

The effective control of infectious diseases, such as COVID-19, depends on an at-risk population’s willingness to engage in coordinated, preventative behavior. Public health officials have used a variety of strategies to increase compliance with recommended public health behaviors, including general education^29,30^, appeals to moral responsibility^9^, fear appeals^31,32^ and behavior modification^33–35^. Despite these efforts, compliance with recommended behaviors, including mask wearing, has been modest in several countries. However, as Cornelia Betsch aptly put it, “in this pandemic, fast and massive behavioral change is key”^15^, p. 438. Only recently have researchers recommended that policy makers draw on the behavioral science to understand the psychological processes that in(de)crease individuals’ motivation to comply with public health mandates. Our research responds to this call by focusing on the link between the need to self-express and public health behaviors.

We content that wearing a face mask may be perceived as restrictive. From a self-expression perspective, a protective face mask might even be seen as inhibiting one’s freedom of speech, in a literal sense (it covers one’s mouth). As noted by the Roman philosopher Seneca: Speech is the mirror of the mind (Imago Animi Sermo Est); it follows that a protective face mask might not only be seen as a means of protection but, also, as a threat to one’s personal freedom. At the extreme, this can result in psychological reactance and a reluctance to wear a face mask in public^36^.

Against this backdrop, our paper investigates a fundamentally different behavior-intervention strategy for increasing compliance with facial mask mandates. In particular, we aligned the desired public health behavior with the individual’s need for self-expression and, consequently, made compliance fun rather than threatening. Three studies validate the idea that facial mask personalization encourages facial mask wearing. More broadly, our findings indicate that health behaviors can be encouraged by making them more representative of the self, so that people want to express the behaviors. This idea can be extended to other preventative health behaviors. For example, hand sanitizer can be customized with personalized packaging or scents. Condom use can be framed as an expression of sexuality, masculinity, or independence instead of safety and disease prevention. Keeping sick children home from school can be framed as an expression of family identity instead of classroom safety. Across all of these examples, socially responsible health behaviors are more likely to be expressed when they signal a valued part of one’s identity.

Despite the importance of our findings, our studies are not without limitations. First, the studies only investigate facial mask wearing. They do not investigate other protective behaviors against COVID-19. Second, only study 1 uses a nationally representative sample. Studies 2 and 3 use convenience samples, the implication being that the direction of the observed experimental effect, not the size of the observed experimental effect, should replicate in other contexts/countries. Third, while evidence supporting the effectiveness of facial mask wearing is compelling^2–8^, there is little research on the effects of mask policies on compliance with other preventive health behaviors (e.g., physical distancing, vaccination). Wearing a face mask might give people a false sense of security and decrease their compliance with other pandemic-mitigation behaviors. While the initial evidence is inconsistent with the hypothesis that mask mandates lead to decreases in physical distancing^37^, this conclusion may change when mask customization is the source of increased compliance. It is possible that self-customized masks communicate individuality which, in turn, maximizes perceived social distance and, consequently, increases the actual physical distance among people^38^. Fourth, our research does not account for possible variations in the need to self-express, which may vary by individual or within different contexts/cultures^23^. In our research contexts, customization was a positive form of expression, which encouraged mask wearing. In other contexts, customization could be a negative form of expression (e.g., customization that emphasizes group-identity over personal identity), which could discourage mask wearing. Consequently, future research needs to look at how incorporating self-expression into specific preventative behaviors interacts with contextual factors to influence the entire corpus of preventative health behaviors.

Despite these limitations, our research provides a novel approach to increasing compliance with public health recommendations. In times when compliance fatigue seems omnipresent, we need to think outside the box when trying to nudge people to stay—or get—on track. We believe that a promising public policy strategy is to frame preventative health behaviors as consistent with citizens’ individual needs.

## Supplementary Information

Supplementary Information.

## Data Availability

All data relevant for this research can be accessed via the following link: https://osf.io/wjgcp/?view_only=04f8f5324fe5474f934b7af4df085f10.
