# Author Correction: Facial mask personalization encourages facial mask wearing in times of COVID-19

**DOI:** 10.1038/s41598-022-07231-2

**Published:** 2022-02-22

**Authors:** Johanna Palcu, Martin Schreier, Chris Janiszewski

**Affiliations:** 1 Department of Marketing, Vienna University of Economics and Business (WU), Vienna, Austria; 2 Warrington College of Business, University of Florida, Gainesville, FL USA

Correction to: *Scientific Reports* 10.1038/s41598-021-04681-y, published online 18 January 2022

The original version of this Article contained an error in the order of the References 36 and 37. References 36 and 37 were published as References 37 and 36.

The correct order of the references is listed below:

36. Taylor, S. & Asmundson, G. J. Negative attitudes about facemasks during the COVID-19 pandemic: The dual importance of perceived ineffectiveness and psychological reactance. *PLoS ONE* **16**, e0246317 (2021).

37. Seres, G., Balleyer, A.H., Cerutti, N., Friedrichsen, J. & Süer, M. Face mask use and physical distancing before and after mandatory masking: No evidence on risk compensation in public waiting lines. 10.2139/ssrn.3924790 (2021).

The original Article has been corrected.

