# Supplementary material for: Facial mask personalization encourages facial mask wearing in times of COVID-19
Source: Sci Rep. 2022 Jan 18;12:891. doi: 10.1038/s41598-021-04681-y (PMC8766589; doi:10.1038/s41598-021-04681-y)
Supplement: Supplementary file 1 — Supplementary Information. [file 41598_2021_4681_MOESM1_ESM.docx]

Supplementary Materials for

**Facial Mask Personalization Encourages Facial Mask Wearing In Times of COVID-19**

**Authors:** Johanna Palcu^1*^, Martin Schreier^1^, Chris Janiszewski^2^

**Affiliations:**

^1^Vienna University of Business and Economics (WU), Austria.

^2^Warrington College of Business, University of Florida, FL, USA.

*Correspondence to: johanna.palcu@wu.ac.at

**This PDF file includes:**

Materials and Methods

Figs. S1 to S11

Tables S1 to S8


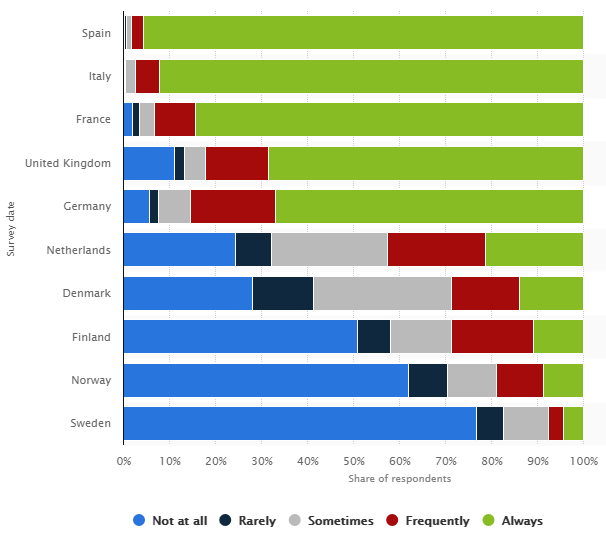


**Figure S1:** Mask wearing compliance in EU countries (*1*). *Source*: Imperial College London. How often have you worn a face mask outside your home to protect yourself or others from coronavirus (COVID-19)? (as of January 10, 2021) *Statista*. <https://www.statista.com/statistics/1114375/wearing-a-face-mask-outside-in-european-countries/> (2021).

**Materials and Methods**

Study 1: Correlational Study

**Table S1.** Survey and sample information (Study 1)

| *Data collection period* | | |  |
| --- | --- | --- | --- |
|  | May 2020^*^ | | |
|  | *Study information*  This study was part of a large-scale online survey on consumer behavior during the COVID-19 pandemic. The study was conducted by a professional marketing research agency with a nationally representative sample. The sample frame (list of potential participants) and sample draw (participants who were contacted) were determined by the marketing research agency based on national quotas. Given the general framing of our research (COVID-19), self-selection based on interest for the specific topic of mask wearing is unlikely. Each participant was assigned a unique code to obviate duplicates during data collection. The study included key outcomes such as intentions to wear a mask, changes in consumers’ purchasing behavior during the pandemic, and tracking app acceptance. The survey was distributed in two waves (with a 10-day time period in-between waves). The intention to wear a mask was most germane to this research. | | |
|  |  | | |
| *Sample statistics* | | |  |
|  | Age group | *n* | Gender distribution (% female) |
|  | 18‒24 | 121 | 62% |
|  | 25‒34 | 170 | 52% |
|  | 35‒44 | 201 | 48% |
|  | 45‒54 | 269 | 58% |
|  | 55-64 | 265 | 55% |
|  | 64-99 | 196 | 30% |
|  | ***M* = 47.63 (*SD* = 15.87)** | ***N* = 1224** | **51%** |

*Note: The study was distributed during the first nationwide lockdown. During this time, strict COVID-19 regulations were in place in the country (i.e., only grocery stores and public offices were open to consumers and protective masks were mandatory in all stores, public buildings, and on public transportation).

**Table S2.** Measures assessed in the first wave in order of presentation (**Measures critical to Study 1 are in bold).**

| **Measure** | | **Coding** |
| --- | --- | --- |
| **First wave** | |  |
| COVID-19 Tracking App General |  | no (1)/yes (2) |
| *Do you own a smartphone?*  *Do you know the STOPP Corona App^1^ of the Austrian Red Cross?*  *If yes, have you downloaded the STOPP Corona App of the Red Cross?* | |  |
| **COVID-19 Risk Perception** | | **Cronbach’s α = .82** |
| ***I feel that the COVID-19 crisis is a severe threat to us.***  ***The current COVID-19 situation makes me feel worried.***  ***I feel that the crisis around COVID-19 is overblown. (r)*** | | **totally disagree (1);**  **totally agree (5)** |
| COVID-19 Tracking App Information^2^ | |  |
| *How likely is it that you will download the STOPP Corona app in the next few days?*  *How likely are you to recommend the STOPP Corona app to your friends and family in the next few days?* | | not at all likely (1);  very much likely (7) |
| Collective Efficacy (*2*) | |  |
| *How confident are you that together we Austrians can reach the following outcomes with the STOPP Corona app?*  *Minimize the likelihood for infections*  *Slow the spread of the COVID-19 pandemic*  *Relieve the health care system*  *Help resolve the COVID-19 crisis* | | not at all confident (1);  very much confident (7) |
| Future Norms (*3*) | |  |
| *How strongly do you believe, that in the foreseeable future, many people will make an effort to download the Stopp Corona App?*  *To what extent do you believe that many Austrians will download the Stopp Corona App in the upcoming days?*  *How strongly do you think that the number of downloads will rise in the upcoming days?* | | not at all (1);  somewhat(4);  very much (7) |
| **Gender**  **Age** | |  |

^1^ COVID-19 tracking app is currently utilized in Austria;

^2^The first wave of this large-scale survey included a study in which we manipulated the information on the tracking app STOPP Corona and measured participants’ intention to download the app.

**Table S3.** Measures assessed in the second wave in order of presentation (unrelated to present research)

| **Measure** | Coding |
| --- | --- |
| **Second wave (10-days later)** |  |
| COVID-19 Tracking App Behavior^1^ |  |
| *Did you download the Stopp Corona App after the last survey?*  *Did you recommend the Stopp Corona App to your friends or family after the last survey?* | no (1)/yes (2) |
| *When did you download the app?* | immediately after the survey (1);  today(7) |
| Self-/Other-Benefit (*4*) |  |
| *To what degree does using this app serve an altruistic goal [i.e., focused on helping others]?*  *To what degree does using this app serve an egoistic goal [i.e., focused on helping oneself]?* | not at all (1);  very much (7) |
| Individualistic Self-construal (*5*)^2^ |  |
| *I enjoy being unique and different from others in many ways.*  *I often do “my own thing”.*  *I am a unique individual.* | not at all (1);  very much (7) |
| Collectivistic Self-construal (*5*)^3^ |  |
| *My happiness depends very much on the happiness of those around me.*  *I often have the feeling that my relationships with others are more important than my own accomplishments.*  *If someone from my Austria got a prize, I would feel proud.*  *To me, pleasure is spending time with others.*  *The well-being of my fellow citizens (Austrians) is important to me. I feel good when I cooperate with others.* | not at all (1);  very much (7) |
| Privacy Concerns |  |
| *While thinking about downloading the Stopp Corona app, I was worried about my privacy.* | totally disagree (1);  totally agree (7) |
| **Transition to Mask Survey^4^** |  |

Note: Measures that are relevant for the present research are highlighted in bold.

^1^The first wave of this large-scale survey included a study in which we manipulated the information on the tracking app STOPP Corona and measured participants’ actual download behavior (second wave).

^2,3^Individualistic and collectivistic mindsets were assessed as part of a different research project.

^4^To separate the survey on the Stopp Corona App from the survey on protective masks we included the following transition between questions: “Thank you for your feedback on the Stopp Corona App. In the following, we would like to ask you some questions on another topic, namely protective masks. Since the beginning of April wearing a protective mask is mandatory in all stores, in public buildings, and on public transportation. In the following, we would like to know more about how you are personally affected by this regulation.”

**Table S4.** Measures assessed in the second wave in order of presentation (**Measures critical to Study 1 are in bold).**

| **Measure** | Coding |
| --- | --- |
| **Mask Wearing** |  |
| ***Please think about how often you have worn your mask over the past few days. Please indicate how frequently you have worn the mask.*** | **only when I really had to (e.g. in the store or public transportation) (1); as frequently as possible (e.g. when leaving the house) (7)** |
| ***There are several different types of masks available (from typical low-budget medical protective masks to do-it-yourself masks). Please describe the mask that you are wearing most frequently.*** | **Open** |
| ***Think about the mask that you wear most often. Where would you place that mask on a scale from a typical mask to a very individual mask?*** | **1= typical; 6 = individual** |
| *Is your mask self-made or bought?* | self-made (1)/bought (2) |
| Consumer Purchasing Behavior^1^ |  |
| *We would now like to know how your purchasing behavior has changed since the beginning of the COVID-19 pandemic.*  *Buying traditional products*  *Spontaneously buying products I hadn’t planned to buy*  *Buying well-known products and brands*  *Buying sustainable products (i.e. products that do not damage the environment)*  *Buying regional products*  *Buying at low prices*  *Trying out new brands and products*  *Buying products that tell something about myself*  *Treat myself with some luxury*  *Buying healthy products*  *Watching out for special promotions* | much less than before (1), same as before (4), much more than before (7) |
| Identity Threat | Cronbach’s α = .85 |
| *With the mask I feel robbed of my identity.*  *When wearing the mask I feel like one out of many.*  *When wearing the mask I feel like I would somewhat get lost in the masses* | totally disagree (1);  totally agree (7) |

| **Mask Restrictedness** | **Cronbach’s α = .70** |
| --- | --- |
| ***When waring the mask I feel that I have everything under control.***  ***I feel physically restricted when wearing the mask.***  ***With my mask, I feel that I am not “functioning” properly (i.e. my senses are restricted).*** | **totally disagree (1);**  **totally agree (7)** |

^1^Consumers’ purchasing behavior was assessed as part of a different research project.

Study 2: Influence of personalizing mask

**Table S5.** Study and sample information (Study 2)

| *Data collection period* | | |  |
| --- | --- | --- | --- |
|  | December 2020^*^ | | |
|  | *Study information*  An online study was conducted using the research platform Prolific. It was a convenience sample. Participants were invited to participate using a general shopping frame (“a study on shopping behavior”) to avoid self-selection based on the topic of mask wearing. Participants received payments of 0.75 £ for their participation. Data was collected in two waves. | | |
|  | *Design*  The study used a mixed-factor design with mask frequency measured at two points-in-time (three day delay in-between measurements) and mask individuality manipulated between-subjects at the second point-in-time (Factor: mask individuality). | | |
|  |  | | |
| *Sample statistics* | | |  |
|  |  | Age | Gender (% female) |
|  | **N = 895** | ***M* = 38.71 (*SD* = 13.59)** | **59.3%** |

*Note: The study was distributed while strict COVID-19 regulations were in place in the country of study (i.e., protective masks were mandatory in all stores, in public buildings, and on public transportation).

**Figure S2***.* Study instructions for control condition for the second wave (Study 2).

*Consider the following:*

*According to government regulations wearing a protective mask****has become voluntary when outside****but****mandatory in most indoor places and on public transportation.****You therefore decide to go to the next available store and buy a new mask.*

-page break-

*In the store they have a set of the masks you see below.*

*
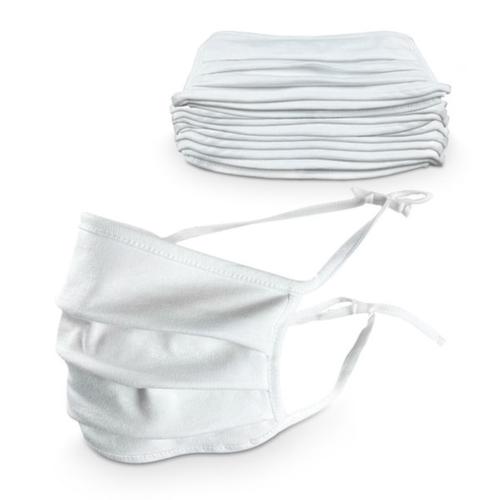
*

-page break-

You pick up one of the masks and head for the checkout.

**Figure S3***.* Study instructions for personalization condition for the second wave (Study 2).

*The store also offers the possibility to customize your mask. They have several options to pick from:*

*They have****numerous patches****, small and large ones, some are colorful and some are black and white. Some show famous icons and bands, hobbies and activities, both usual and unusual, a huge selection of food pictures (from strawberries to avocados, from burgers to pho), your favorite movies and TV shows (E.T. is calling), pictures of famous artists, political statements and much more. You can either pick one of the patches they have in the box or they can print out whatever patch you have in mind. You name it.*

*Also, they have****letters that can be printed on the mask****, if you want to include your initials or a message of your choice. Again you can express whatever you like. You can make bold statements, share how you're feeling or simply put your initials on the mask. If you want to keep it private, you can even add the message or your initial on the back of the mask.*

*Finally, you can also pick between****colorful rubber-bands****that you can exchange for the white bands that are on the mask.*

-page break-


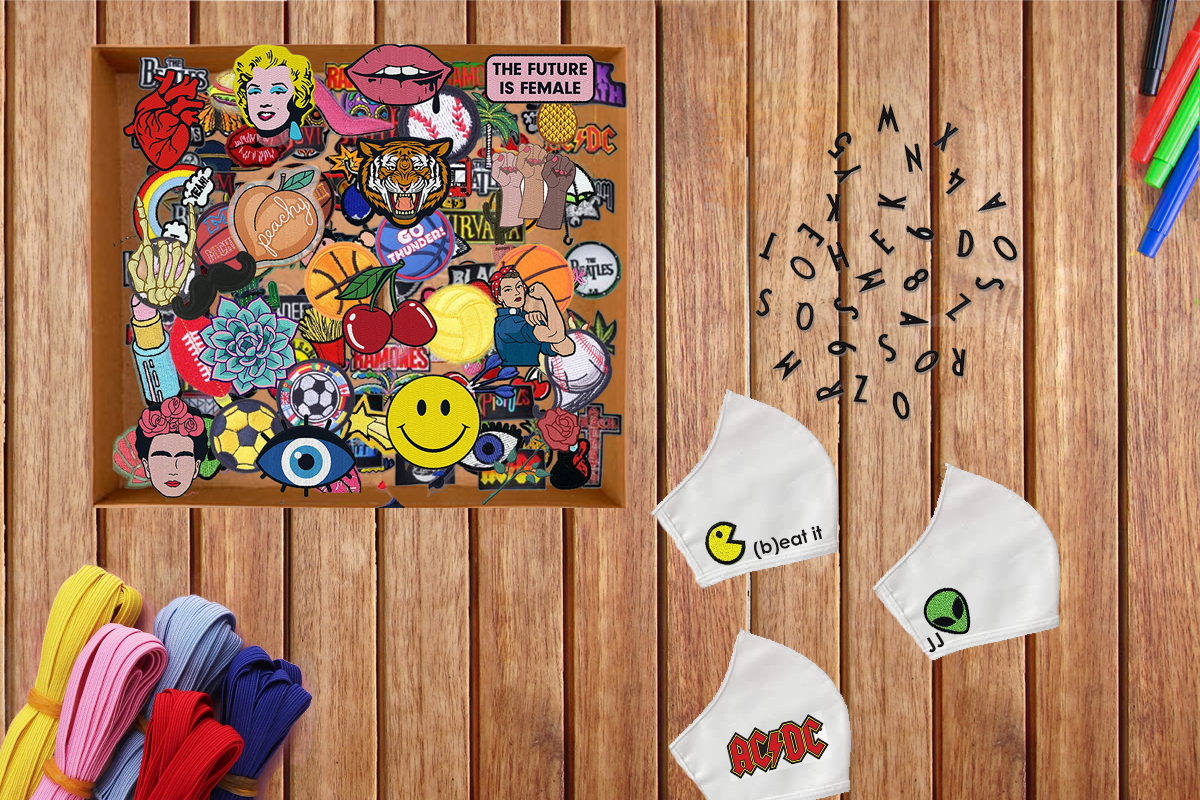


*With these tools you can customize the mask you wish. How would your personal mask look like?****Please briefly describe what you would put on your mask in just a few words:*** (open response)

-page break-

Once you are ready you pick up your self-customized mask and head for the checkout.

**Table S6.** Measures in order of presentation (Study 2)

| **Measure** | | **Coding** |
| --- | --- | --- |
| Mask Wearing Intensity^1,2^ |  | only when really necessary (e.g., in public transportation or indoors) (1); whenever I can (e.g., as soon as I leave the store) (7) |
| *How frequently do you think you will wear the mask that you just picked?* | |  |
| Mask Self-Expressiveness^2^ | | r = .91 |
| *This mask allows me to express my identity.*  *This mask helps me signal who I am.* | | totally disagree (1);  totally agree (7) |
| Need for self-expression (*6*)^1^ | | Cronbach’s α = .77 |
| *In general, I would like to be perceived as different from the general population.*  *I often purchase products that let me express my uniqueness.*  *It is important for me to be able to express my identity.* | | totally disagree (1);  totally agree (7) |
| COVID-19 Risk Perception^1^ | | Cronbach’s α = .86 |
| *I feel that the COVID-19 crisis is a severe threat.*  *The current COVID-19 situation makes me feel worried.*  *I feel that the crisis around COVID-19 is overblown. (r)* | | totally disagree (1);  totally agree (5) |
| Gender  Age | |  |

^1,2^Numbers indicate whether measure was assessed during first (1) or second (2) wave.

Study 3: Field Study

**Table S7.** Study and sample information (Study 3)

| *Data collection period* | | |  |
| --- | --- | --- | --- |
|  | October 2020^*^ | | |
|  | *Study information*  A field study was conducted on a University Campus (subject pool of the University). Participants were recruited for the university subject pool using campus flyers. Participants were invited to participate using a general shopping frame (“Participate in an on-campus study on shopping behavior”) to avoid self-selection based on the topic of mask wearing. Participants received monetary compensation for their participation (10 Euro). | | |
|  | *Design*  One-factorial between subjects design (Factor: mask individuality) | | |
|  |  | | |
| *Sample statistics* | | |  |
|  |  | Age | Gender (% female) |
|  | **N = 99** | ***M* = 23.13 (*SD* = 2.30)** | **73%** |

*Note: The study was run just before the second nationwide lockdown. During this time, rather strict COVID-19 regulations were in place in the country of study (i.e., all stores and public offices were open to consumers and protective masks were mandatory in all stores, in public buildings, and on public transportation).

Figure S4. Study flow (Study 3)

Figure S5. Introductory study instructions regardless of condition (Study 3)

*Welcome and thank you for participating in our studies. Before we start, can I please ask you to use the disinfectant to clean your hands? Thank you!*

*Today you will be participating in two different studies. The first study is about how consumers design masks and the second study is a short Mystery Shopping task. Finally, at the end of the session, we would like to ask you to fill out a short questionnaire in the LAB. Are you ready to start?*

**Figure S6**. Study instructions for customization task in customization condition (Study 3)

*For the first study, please follow me to the next room where I will explain the task in more detail.*

*In the first study, we would like to know more about how consumers design protective face masks. We will first ask you to design your own personal mask. We will ask you a few short questions about the process at the end of the session. On the table, you see a white mask and a few tools that you can use to design your own individual mask. Here you have patches that you can put on your mask as well as a few pens, if you want to write a message on your mask or sign it. Before you start, take your time to look at the samples that you see on the table. You can use these masks as an inspiration or come up with something different that you like and that reflects your personal style. Before you start, here is some advice and ground rules (you can also find these on the paper in front of you if you want to read them again):*

*You can (but you don’t have to) use a maximum of 3 patches. Some people like to be subtle and prefer smaller patches or no patch at all, others like it more colorful and prefer to use many patches. Do whatever suits you best. But keep in mind to use a maximum of 3 patches.*

*You can use the pens and/or the stamps in front of you to sign your mask or write a message on your mask. Just apply some color with the pens or the stamps if you want to use those. You can go colorful or use one color only or not use the pens at all, whatever you prefer. Again, some people would choose to write their name or something personal on the inside of the mask (something only they would know). Others would prefer to write something on the outside (something others would see). You could, for instance, write on the rubber band or directly onto the mask. Whatever you prefer. One advice though: Try not to go crazy and “overdesign”. You should create something that you like and that reflects your personal style.*

*Are you ready? Ok, you can start designing your personal mask. I will wait outside. Once you are ready please spray some of the odorless disinfectant onto the mask and then come outside. I will be then taking a picture of your mask*

*.*

**Figure S7**. Study instructions for purported second study (Mystery shopping task; Study 3)

*Thank you for participating in the first study. In the second study we are interested in finding out how service encounters have changed during times of COVID-19. So for the second study, we will send you on a short mystery shopping trip to the bakery on campus. The employees at Anker were not informed that you will be going there on our behalf.*

*In this envelope you have two Euros. Your task will be to go into the bakery on campus and buy something for yourself. Please have a look at the products and pay attention to the service encounter and the ambience at the bakery. You should buy at least one item. You can buy whatever you like and as many products as you want. You can use up all money or even add some of your own money if you want to buy more. Please keep the receipt after you have paid and then leave the store. After you leave the bakery, one of our students will be waiting outside, wearing a blue name tag with the University logo. Please hand her your receipt and she will ask you some short questions.*

*Do you have any questions?*

[High mask individuality condition]: *We want to make sure that the contextual factors are similar for all participants so* ***we would like to ask you to wear the mask that you just designed****. Please put the mask on. Here’s a mirror. Is the mask fitting ok or do you want to adjust something?*

[Low mask individuality condition]: *We want to make sure that the contextual factors are similar for all participants so* ***we would like to ask you to wear one of our masks* (hand over white mask**). *Please put the mask on. Here’s a mirror. Is the mask fitting ok or do you want to adjust something?*

*Before you start:* ***Don’t forget that according to campus regulations it is mandatory to wear a mask inside the buildings (that is inside the LAB) and when you’re inside the store. When you’re outside you can take the mask off if you like, or you can leave it on whatever you prefer.***

**Figure S8**. Exemplary masks in the experimental conditions of Study 3 (left: self-customized by participant; right: control mask)


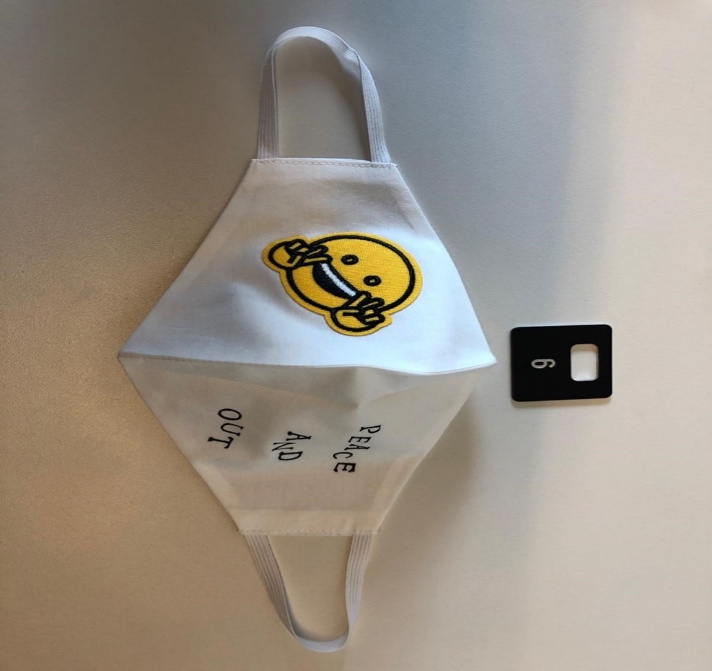

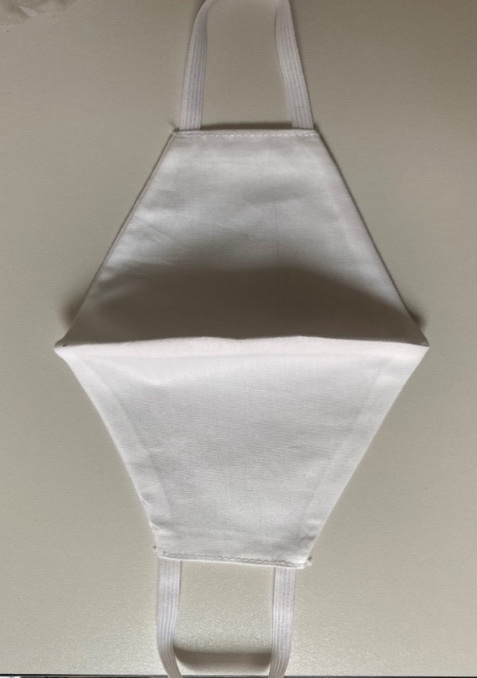


Figure S9. Set-up for customization task (Study 3)


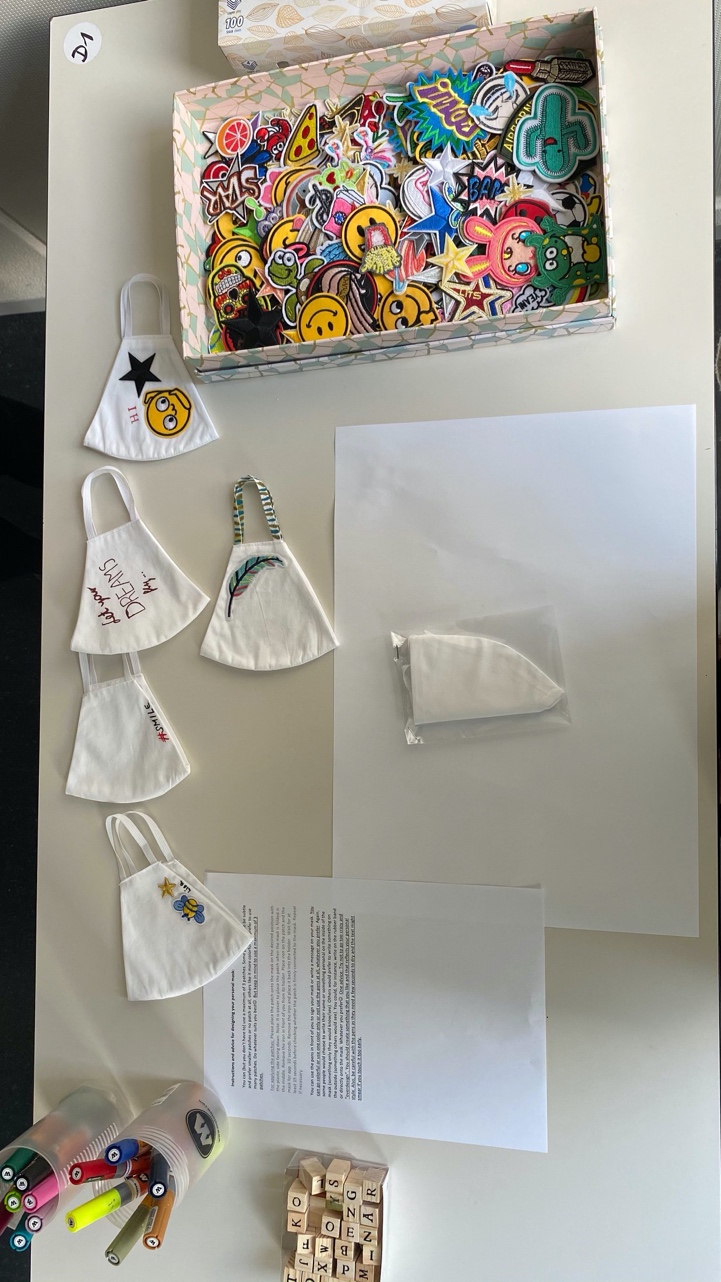


**Figure S10.** Set-up for observations (Study 3)

**
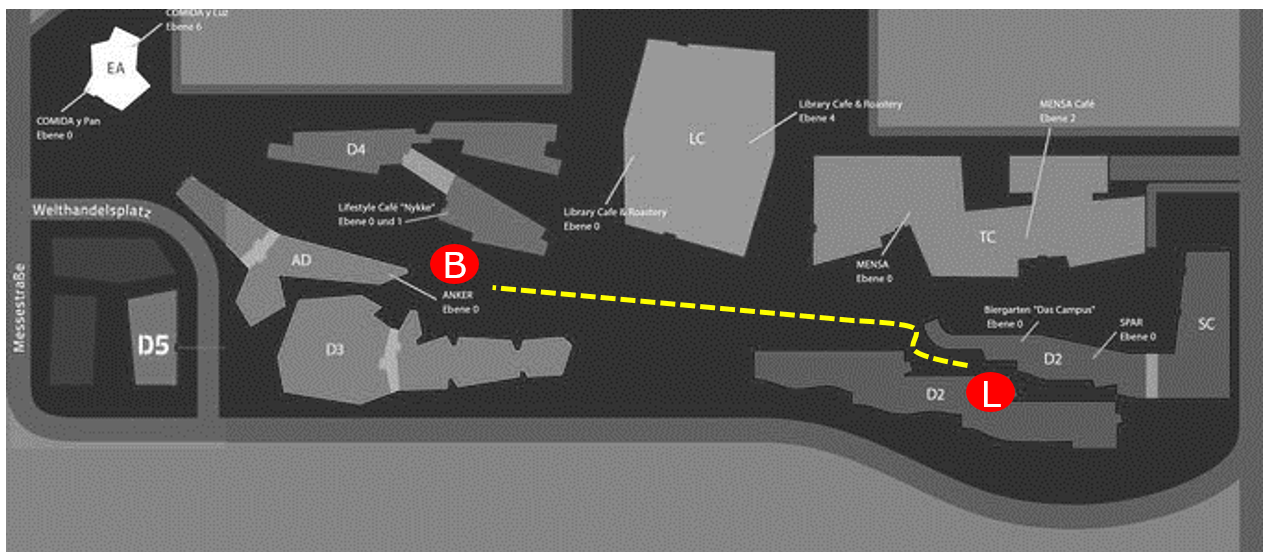
**

Mask wearing was voluntary from the laboratory to the bakery (track marked in yellow). We observed whether participants wore the mask on five different occasions: when they left the lab (L), when they arrived at the bakery (B), when they left the bakery, when they talked to the experimenter located outside the bakery, and when they arrived back at the lab.

**Figure S11.** Observation sheet for research assistant positioned at the lab (Study 3).

| Mask wearing (Coding in brackets) | Notes: |
| --- | --- |
| **Mask wearing when leaving LAB:**  O Mask is off before leaving LAB door (inside LAB) (1)  O Mask is off directly after leaving LAB door (outside LAB)(2)  O Mask is off after a few steps (Outside LAB) (3)  O Doesn’t take mask off (4) |  |
| **Mask wearing when returning to LAB:**  O Mask is on the whole time (4)  O Mask is on a few steps before lab (outside the LAB) (3)  O Mask is on shortly before entering LAB (outside the LAB) (2)  O Mask is on after entering LAB (inside the LAB) (1) |  |

Note: Research assistants were blind to the purpose of the study. All research assistants were informed that observations were made in order to control for possible effects of mask wearing on shopping behavior.

**Figure S12.** Observation sheet for research assistant positioned at the bakery (Study 3).

| Mask wearing (Coding in brackets) | Notes: |
| --- | --- |
| **Mask wearing when arriving at BAKERY:**  O Wears mask the whole time (4)  O Puts mask on a few steps before BAKERY (3)  O Puts mask on shortly before entering BAKERY (outside of BAKERY) (2)  O Puts mask on after entering BAKERY (inside BAKERY) (1) |  |
| **Mask wearing when leaving BAKERY:**  O Takes mask off before leaving BAKERY door (still inside BAKERY) (1)  O Takes mask off after leaving BAKERY door (when outside BAKERY) (2)  O Takes mask off after a few steps outside (3)  O Doesn’t take mask off (4) |  |
| **Mask while talking to experimenter:**  O has mask on (2)  O doesn’t have mask on (1) |  |

Notes: (a) Research assistants were blind to the purpose of the study. All research assistants were informed that observations were made in order to control for possible effects of mask wearing on shopping behavior; (b) The research assistant at the bakery was instructed to hide when participants arrived at the bakery. After the participant had left the bakery, the research assistant assessed the participants’ shopping experience (ambience, smell, sound, customer service, product range; 1= *very good*/5 = *very bad*). This procedure helped disguise the actual purpose of the study; (c) We also assessed the number of customers present at the bakery (1 = *very few*; 5 = *many* (10 or more)).

**Table S8.** Measures in online questionnaire in order of presentation (Study 3)

| **Measure** | | **Coding** |
| --- | --- | --- |
| Joy |  | totally disagree (1);  totally agree (7) |
| *I enjoyed wearing the mask.* | |  |
| Physical Restrictedness | |  |
| *I felt physically restricted (e.g. limited smell) while wearing the mask.* | | totally disagree (1);  totally agree (7) |
| Mask Self-Expressiveness | | Cronbach’s α = .89 |
| *This mask allows me to express my identity.*  *This mask helps me signal who I am.* | | totally disagree (1);  totally agree (7) |
| Identity Threat | | Cronbach’s α = .71 |
| *With the mask I feel robbed of my identity.*  *When wearing the mask I feel like one out of many.*  *When wearing the mask I feel like I would somewhat get lost in the masses* | | totally disagree (1);  totally agree (7) |
| COVID-19 Risk Perception | | Cronbach’s α = .60 |
| *I feel that the COVID-19 crisis is a severe threat to us.*  *The current COVID-19 situation makes me feel worried.*  *I feel that the crisis around COVID-19 is overblown. (r)* | | totally disagree (1);  totally agree (5) |
| Gender  Age | |  |

References

1. Imperial College London. How often have you worn a face mask outside your home to protect yourself or others from coronavirus (COVID-19)? (as of January 10, 2021) *Statista*. <https://www.statista.com/statistics/1114375/wearing-a-face-mask-outside-in-european-countries/> (2021).
2. Bandura, A. Guide for constructing self-efficacy scales. *Self-efficacy beliefs of adolescents* **5**, 307-337 (2019).
3. Sparkman, G. & Walton, G.M. Dynamic norms promote sustainable behavior, even if it is counternormative. *Psych Sci* **28,** 1663-1674 (2017).
4. White, K. & Peloza, J. Self-benefit versus other-benefit marketing appeals: Their effectiveness in generating charitable support. *J Mark* **73**, 109-124 (2009).
5. Brewer, M. B. & Chen, Y. R. Where (who) are collectives in collectivism? Toward conceptual clarification of individualism and collectivism. *Psych Rev* **114**, 133 (2007).
6. Chernev, A., Hamilton, R. & Gal, D. Competing for consumer identity: Limits to self-expression and the perils of lifestyle branding. *J Mark* **75,** 66-82 (2011).

**Data source:**

All data critical for this research can be accessed via the following link: https://osf.io/wjgcp/?view_only=04f8f5324fe5474f934b7af4df085f10
